# Photosynthetic Effect in *Selenastrum capricornutum* Progeny after Carbon-Ion Irradiation

**DOI:** 10.1371/journal.pone.0149381

**Published:** 2016-02-26

**Authors:** Jie Wang, Xin Li, Dong Lu, Yan Du, Liang Ma, Wenjian Li, Jihong Chen, Fuli Li, Yong Fan, Guangrong Hu, Jufang Wang

**Affiliations:** 1 Gansu Key Laboratory of Space Radiobiology & Microbial Resources and Application, Institute of Modern Physics, Chinese Academy of Sciences, Lanzhou, PR China; 2 University of Chinese Academy of Sciences, Beijing, PR China; 3 Shandong Provincial Key Laboratory of Energy Genetics, Qingdao Institute of Bioenergy and Bioprocess Technology, Chinese Academy of Sciences, Qingdao, PR China; CEA-Saclay, FRANCE

## Abstract

A large proportion of mutants with altered pigment features have been obtained via exposure to heavy-ion beams, a technique that is efficient for trait improvement in the breeding of plants and algae. However, little is known about the underlying mechanisms by which the photosynthetic pigments are altered by heavy-ion irradiation. In our study, the photosynthetic characteristics of progenies from carbon-ion irradiated *Selenastrum capricornutum* were investigated. Five progenies deficient in chlorophyll *a* were isolated after carbon-ion exposure. Photosynthetic characteristics, photoprotection capacity and gene expression of the light-harvesting complex in these progenies were further characterized by the measurement of chlorophyll fluorescence parameters (Fv/Fm, ФPSII, NPQ, ETR), the de-epoxidation state of the xanthophyll cycle, the amount of lutein and quantitative real-time PCR. High maximum quantum yield of photosystem II at day 10 and high thermal dissipation ability were observed in progenies #23 and #37 under normal culture condition. Progenies #18, #19 and #20 showed stronger resistance against high levels of light steps than the control group (612–1077 μmol photons m ^-2^ s ^-1^, p< 0.05). The progenies #20 and #23 exhibited strong photoprotection by thermal dissipation and quenching of ^3^Chl* after 24 h of high light treatment. The mRNA levels of *Lhcb5*, *Lhcbm5* and *Lhcbm1* of the light-harvesting complex revealed markedly differential expression in the five progenies irradiated by carbon-ion beams. This work indicates that photosynthetic efficiency, photoprotection ability and the expression of light-harvesting antennae in unicellular green algae can be markedly influenced by irradiation. To our knowledge, this is the first report on changes in the photosynthetic pigments of green algae after treatment with carbon-ion beams.

## Introduction

Heavy-ion beams possess the advantages of higher linear energy transfer (LET) and greater relative biological effectiveness (RBE) compared with conventional radiation such as X–rays and gamma rays [1]. On the cellular scale, DNA double-strand breaks, large deletions and insertions, and chromosomal rearrangements can be efficiently induced by heavy-ion exposure [2]. Therefore, a high mutation rate and a wide range of mutation types are obtained. Mutagenesis by heavy-ion irradiation has been used in plant breeding for decades. A high proportion of mutants deficient in photosynthetic pigments and anthocyanin, even albino plants, are found in plants after heavy-ion exposure. Examples include a color-leaf mutant of *Arabidopsis thaliana* [3], a chlorophyll-deficient mutant of *Oryza sativa* [4], an albino mutant of *Nicotiana tabacum* [5, 6] and a color-leaf mutant of *Tradescantia fluminensis* [7].

The photosynthetic process is strongly responsive to ionizing radiation. Up to now, only a few studies have reported on the influence of ionizing radiation on the functionality of the photosynthetic apparatus [8]. For instance, photosystem II (PSII) was shown to be the specific target of gamma radiation on the fine structure of the photosynthetic complex in cyanobacteria [8]. Angelini *et al*. reported that damage to the photosystem and reduced photosynthetic oxygen evolution occurred after exposure to ionizing radiation [9]. Micco *et al*. showed that pigment-protein complexes, electron transport carriers and enzymes of the carbon reduction cycle could be altered by ionizing radiation [8].

Algae have been recognized for their biotechnological potential in the production of biofuels and high-value products such as carotenoids, fatty acids, and recombinant proteins [10]. Physical mutagenesis has recently been applied to algae breeding for trait improvement. Mutagenesis in algae after heavy-ion exposure has been reported recently; the algae studied include the red alga *Porphyra yezoensis* [11], the unicellular green microalga *Parachlorella kessleri* [12], the high–lipid productivity mutant D90G-19 of *Desmodesmus* sp. S1 and the high triacylglycerol content mutant HP-1 of *Nannochloropsis oceanica* IMET1 [13, 14]. However, little is known about the effects of heavy-ion irradiation on the photosynthetic pigments of algae. Therefore, it appears that investigation of the effects of exposure to heavy-ion irradiation on algae photosynthetic pigments offers a powerful approach to studying the effects of ionizing radiation on photosynthesis which is essential to biomass formation.

*Selenastrum capricornutum*, a crescent-shaped, ubiquitously occurring unicellular green alga that is easily cultivated in fresh water, is an ideal model for common standard toxicity tests and the investigation of photosynthetic pigments after exposure to radiation [15]. Chlorophyll fluorescence analysis is a powerful technique for screening desirable plant traits with physiological responses [16]. For instance, Ralph, et al revealed rapid light curves of Z. *marina* shown an elevated level of non-photochemical quenching (NPQ) under high light conditions (300 μmol photons m^-2^ s^-1^) [17]. NPQ of PSII chlorophyll (Chl) *a* emission is a proxy to measure the thermal dissipation used to defend the plant from injury by exposure to excessive energy [18]. Moreover, pigments such as the xanthophyll cycle (XC) pigments and lutein not only function as light donors and acceptors but also protect higher plants and algae from injury by excessive light energy [19]. For instance, *C*. *reinhardtii* has evolved multiple mechanisms to cope with light stress [20]. After short-term of exposure to high light, rapid and reversible changes occur in XC, most of the XC pool converted to zeaxanthin. Therefore, the xanthophyll cycle pigments analysis was considered as another major index.

Carbon ions are one of the most common heavy ions used for mutation research. In the present study, five *Selenastrum capricornutum* progenies deficient in Chl *a* were screened out by the amount of Chl *a* after carbon-ion irradiation. These progenies were characterized by combine chlorophyll fluorescence analysis *in vivo* and quantification of the xanthophyll cycle pigments and lutein *in vitro*. In addition, gene expression of the light-harvesting complex II (LHCII) at mRNA levels was studied to determine whether mRNA expression had been altered.

## Materials and Methods

### Algal Strain and Culture Conditions

*Selenastrum capricornutum* was obtained from the Freshwater Algae Culture Collection at the Institute of Hydrobiology (FACHB-collection), Wuhan, China. The strain was grown in 250-ml Erlenmeyer flasks containing 150 ml CT medium (provided by the FACHB-collection) at 25±1°C under a 12 h:12 h illumination cycle (fluorescent light of 80–100 μmol photons m^-2^ s^-1^) in a light growth chamber MGC-450 BP model (Yiheng Scientific Instrument Ltd., Shanghai, China). The initial inoculum amount was 6.25% by volume. Optical density (OD) was measured at 680 nm to determine the growth rate and to adjust the cell concentration. When the cells reached the mid-logarithmic growth phase, they were harvested by centrifugation at 4,500×g for 20 min at room temperature, washed and resuspended in sterile water in preparation for carbon-ion irradiation.

### Carbon-Ion Irradiation

After adjustment of the cell concentration to 1×10^6^ cells·mL^-1^, the cell suspension was exposed to carbon-ion (^12^C^6+^) irradiation at doses ranging from 0 to 120 Gy (0, 20, 40, 60, 90 and 120 Gy). Irradiation was conducted at the Heavy-Ion Research Facility in Lanzhou (HIRFL), Institute of Modern Physics, Chinese Academy of Sciences. The initial energy was 80 MeV/u. The average LET was 33 keVμm^-1^. After irradiation, the algal cells were diluted to an appropriate concentration, spread onto CT-agar plates in triplicate, and cultured at 25±1°C under illumination (80–100 μmol photons m^-2^ s^-1^) to form colonies for the isolation of single colony.

### Mutant Isolation and Primary Screening

Colonies derived from the irradiated cells were picked according to the growth rates and colors of the colonies grown on the CT-agar plates. Individual colonies were transferred in 2 ml of CT medium to 24-well microplates several times to obtain purified monoclonal strains for amplification. The amount of initial inoculation with the monoclonal strain was 9% by volume. The algal cells were transferred to 10 ml of CT medium in 15-ml glass tubes used for Chl *a* screening.

### Chlorophyll Quantification

Algal cells in the logarithmic growth phase (2, 4, 6, 8, 10 d) were collected by centrifugation at 4,500×g for 20 min at room temperature. A hot ethanol extraction method was used to extract chlorophyll from the algal cells [21]. Collected algal cells were homogenized and incubated in ethanol solvent at 78°C for 5 minutes. Then the cultures were incubated without illumination in ethanol overnight at room temperature, and centrifuged at 4,500×g for 20 min. The supernatant was transferred to 96-well microplates and measured using a microplate spectrophotometer (Tecan Infinite M200pro, Männedorf, Switzerland) at 470 nm, 649 nm and 665 nm. The Chl *a* content was calculated in μg/L as reference [22].

### Measurement of Chlorophyll Fluorescence Parameters

Chlorophyll fluorescence parameters were determined using Imaging-PAM (Henz-Walz GmbH, Effeltrich, Germany). The progenies and the control group were cultured in a light growth chamber and were collected in growth phase (2, 4, 6, 8, 10, 12 d). Two hundred microliters of algal cell suspension of each progeny were transferred into 96-well microplates in triplicate, and the microplate was putted into the Imaging-PAM. After 8 min of dark adaptation, the cells were exposed to different actinic light (AL) (1–1077 μmol photons m ^-2^ s ^-1^) every 20 s to measure Rapid Light Curve (RLC). Each AL was turned off, and the resulting transient increase in Chl fluorescence was recorded under low measuring light. The initial (F_0_) and maximum (F_m_) fluorescence levels were measured to calculate Fv/Fm (the maximum quantum yield of PSII) [16]. The RLC of NPQ, ФPSII(quantum yield of PSⅡ)and the relative photosynthetic electron transport rate (rETR) were calculated after 12 days cultivation [23]. The parameters were set according to the instructions provided by the manufacturer.

### Xanthophyll Cycle and Lutein Measurement after High Light Exposure

The progenies and the control group in mid-growth phase were cultured under different high light exposure time (300–400 μmol photons m ^-2^ s ^-1^; 0, 2, 6, 12, 24, 48, 72, 96 h). Pigments were extracted as described in the subsection of chlorophyll quantification above. After hot ethanol extraction and overnight incubation, the supernatant was carefully filtered through Sterile Syringe Filter, transferred to high performance liquid chromatography (HPLC) vials and analyzed on an HPLC system (Waters e2695, Milford, MA, USA). The HPLC system was equipped with a PDA detector and a ZORBAX Eclipse XBD-C18 reverse-phase column (4.6 mm × 250 mm, 5 μm diameter particles; Agilent Technologies, Englewood, USA). Chromatographic standards (violaxanthin (Vx), antheraxanthin (Ax), zeaxanthin (Zx) and lutein) were purchased from ChromaDex Corporation (Irvine, CA, United States). For the xanthophyll cycle pigments, the elution gradient was run with eluent (acetonitrile: acetone = 95:5) under isocratic elution conditions in 20 min according to Quaas et al. [24]. For lutein analysis, the elution gradient was run with eluent (100%-85% methanol, 0%-15% acetone) under gradient elution conditions in 15 min [25]. The column temperature during separation was adjusted to 25°C, and the flow velocity was 1 min/mL. Quantification of pigments was performed according to the standard curve of each pigment. The de-epoxidation state (DES) was calculated from the concentration of XC pigments as follows:

DES=(Zx+0.5Ax)/(Vx+Ax+Zx).

### Quantitative Real-Time PCR Analysis

Two-step quantitative real-time PCR (qRT-PCR) was conducted using total RNA from the control group and from the five progenies of *Selenastrum capricornutum*. Algal cells in the exponential growth phase were collected by centrifugation and frozen in liquid nitrogen for RNA extraction. The algal pellets were ground with quartz sand in liquid nitrogen until the cells were completely broken. Total RNA was extracted using the Plant RNA Kit (Omega, Norcross, GA, USA) following the manufacturer’s protocol. The RNA samples were reverse transcribed using a Transcriptor First Strand cDNA Synthesis Kit (Roche, Basle, Switzerland) following the instructions provided in the kit manual. Quantitative real-time PCR was performed using gene-specific primers and FastStart essential DNA green master reagent (Roche, Basle, Switzerland) with a qRT-PCR system (Qiagen, Dusseldorf, Germany) following the manufacturer’s instructions. The 18S gene was used as a reference gene for qRT-PCR; the specific primers were *18S*-for (ATGGAATAACACGATAGGACTCTGG) and *18S*-rev (ACCTCTGA CAATGAAATACGAATGC). The primers for *Lhcbm1* were *Lhcbm1*–for (CTACCTGACTGGCGAGTTCC) and *Lhcbm1*-rev (CCTCCTGGAAGATCTGAGCA). The primers for *Lhcb5* were *Lhcb5*-for (GACCTGGACAAGTG GTACGG) and *Lhcb5*-rev (CAGAGGGTCGTAGCCGTAGT). The primers for *Lhcbm5* were *Lhcbm5*-for (GCTTCTTCGTCCAGGCCATC) and *Lhcbm5*-rev (GAGGGGTGAACTTCTGGGC). Reactions were performed in triplicate for each sample.

### Statistical Analysis

Data from triplicate samples are presented as the mean ± standard deviation. The significant differences between the means were tested using ONE-WAY ANOVA in the SPSS statistical package (version 17.0; SPSS Inc., Chicago, IL, USA) at a significance level of *p* < 0.05.

## Results

### Survival and Chlorophyll *a* Screening of Progenies from *Selenastrum capricornutum* after Carbon-Ion Irradiation

**Fig 1:**
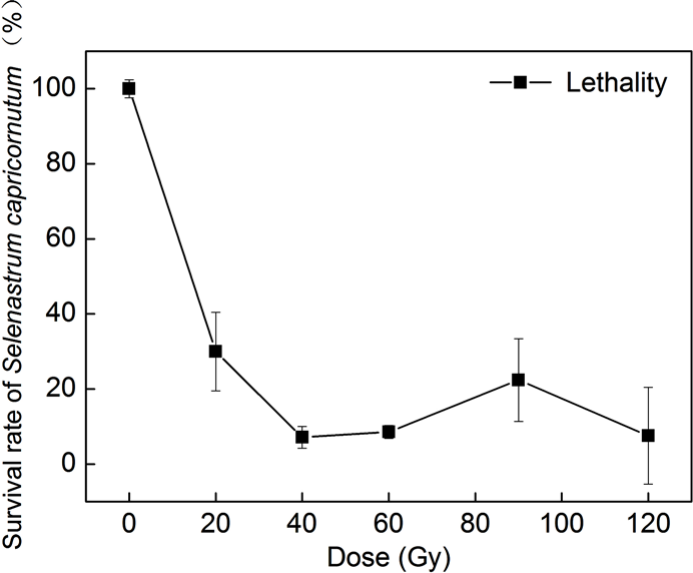
Survival rate of *Selenastrum capricornutum* after various doses of carbon-ion irradiation. n = 3±SD.

A colony-forming unit assay was used to measure algal cell lethality after carbon-ion irradiation. As shown in Fig 1, the survival rate of *Selenastrum capricornutum* decreased as the radiation dose increased. When the dose exceeded 40 Gy, the survival rate decreased to 10%. Our results indicate that *Selenastrum capricornutum* is more sensitive to carbon-ion beams than are other unicellular green alga [12, 13, 14].

**Fig 2:**
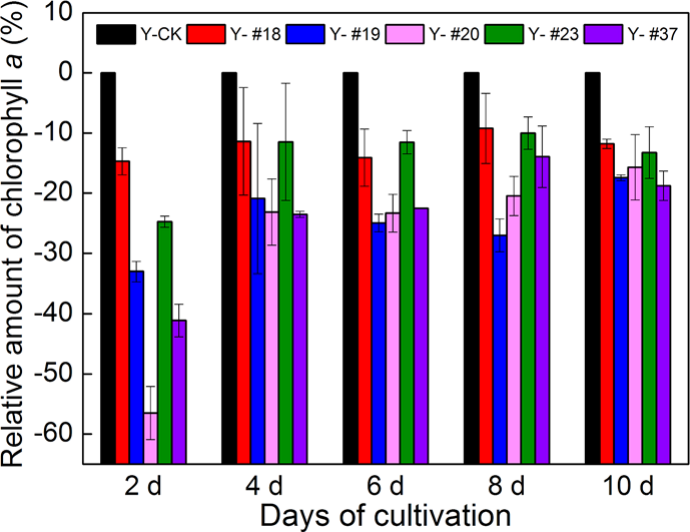
Screening of *Selenastrum capricornutum* progenies based on the relative content of Chl *a*. The amount of Chl *a* in each progeny was compared with the amount present in the control group(Y-CK) at each time point. The initial inoculation amount of each progeny and of the control group was 6.25% by volume. OD 680 nm of the progenies and of the control group (0 d) was measured, and the Chl *a* measurement was not influenced by the growth rates. The error bars indicate the SD of six biological replicates from two independent screenings.

Progenies from irradiated *Selenastrum capricornutum* were chosen visually according to their sizes and colors from numerous colonies grown on CT-agar plates and were transferred to 24-well microplates for amplification. Chl *a* content was chosen as the screening index for progenies. After two rounds of screening, five progenies deficient in Chl *a* were obtained (Fig 2). The Chl *a* content of the five progenies was lower than that of the control group at time points measured (2, 4, 6, 8, and 10 d; S1 Table, p< 0.05), especially at day 2 (14.7%-56.5% reduction). Although, Chl *a* content in the five progenies increased gradually during the 10-day cultivation period (S1 Table), the relative content of Chl *a* in the progenies remained lower than that of the control group (11.8%-18.8% reduction).

### Characterization of the Chlorophyll Fluorescence Parameters of Progenies Cultured under Normal Condition

**Fig 3:**
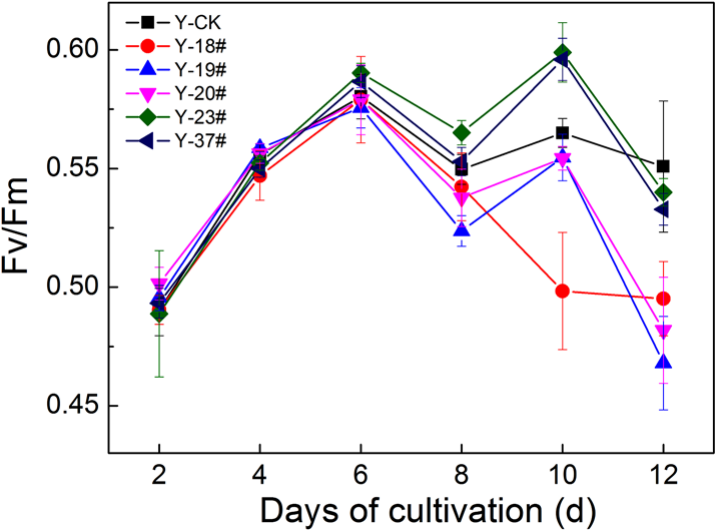
Fv/Fm of PSII of the progenies and of the control group. The Fv/Fm of the control group and of the progenies was determined at the indicated time points after dark adaptation for 8 min. Minimal fluorescence in the dark and maximal fluorescence upon a saturating light pulse were measured by Maxi-IMAGING-PAM at room temperature. The initial inoculation amount of each progeny and of the control group was 6.25% by volume; the Fv/Fm of the initial algal cell cultures (0 d) was too low to measure. n = 3+SD.

As shown in Fig 3, the Fv/Fm values of progenies #18, #19, #20 and that of the control group increased to a maximum (0.57–0.58) on the 6th day. The Fv/Fm values of progenies #23 and #37 reached their highest levels (0.596–0.599) on the 10th day. In contrast to the control group, progenies #23 and #37 showed high maximum quantum efficiency of PSII on day 10 (p< 0.05), and progenies #18, #19 and #20 revealed low Fv/Fm values on days 8 and 12 (p< 0.05).

**Fig 4:**
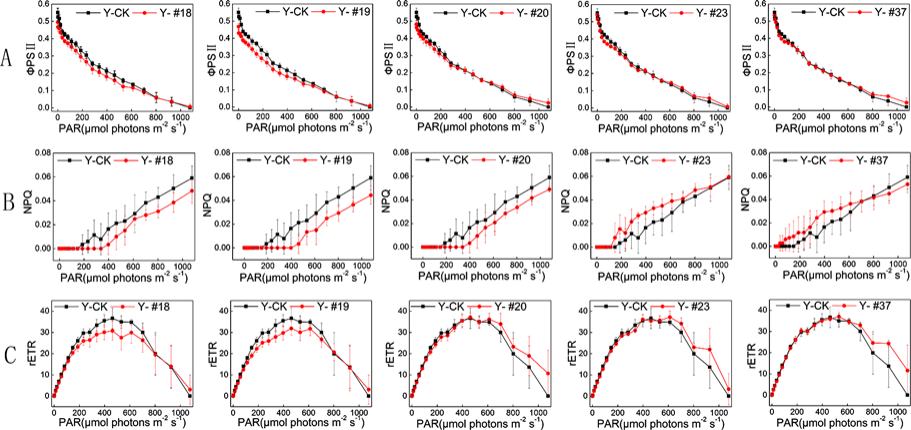
Chlorophyll fluorescence parameters of the progenies and of the control group. A: RLC of PSII quantum yield of the five progenies and of the control group. B: RLC of NPQ of the five progenies and of the control group. C: RLC of rETR of the five progenies and of the control group. The algal cells were exposed to different AL (1–1077 μmol photons m ^-2^ s ^-1^). Measurements were performed in darkness before at room temperature, and each light step was applied every 20 s. OD 680 nm of the five progenies and of the control group showed no significant differences on the 12th day of cultivation (p < 0.05). n = 3+SD.

PSII quantum yield (ФPSII) indicates the actual photosynthetic efficiency of PSII. As shown in Fig 4(A), the RLC of the ФPSII values of the five progenies and of the control group decreased with increasing photosynthetically active radiation (PAR, 0–1077 photons μmol m ^-2^ s ^-1^). The ФPSII values of progenies #18 and #19 were lower than that of the control group over the light steps range 2–612 μmol photons m ^-2^ s ^-1^ (p< 0.05). In addition, low photosynthetic efficiency at low light steps (12–147 μmol photons m ^-2^ s ^-1^, p< 0.05) was detected for progenies #20, #23 and #37.

NPQ is a protection process that thermally dissipates excess light energy that is not used for photosynthesis [23]. As shown in Fig 4(B), the RLC of the NPQ values of the five progenies and of the control group increased with increasing PAR. The NPQ values of progenies #23 and #37 were higher than that of the control group over the light steps ranges 147–232 μmol m ^-2^ s ^-1^ and 37–232 μmol photons m ^-2^ s ^-1^, respectively (p< 0.05). Progenies #18, #19 and #20 exhibited less thermal dissipation than the control group at high levels of light steps (612–1077 μmol photons m ^-2^ s ^-1^, p< 0.05).

rETR is an approximation of the rate of electrons pumped through the photosynthetic chain [17]. As shown in Fig 4(C), the rETR of the five progenies and of the control group increased in response to increasing PAR and reached a maximum at 462 (progenies #18, #19, #20 and the control group), 612 (#23) or 532 μmol photons m ^-2^ s ^-1^ (#37). The rETR values of progenies #18 and #19 were lower than that of the control group over the light steps range 2–702 μmol photons m ^-2^ s ^-1^ (p< 0.05). Progenies 20#, 23# and 37# showed lower photosynthetic electron transport rates than the control group at low levels of light steps (12–147 μmol photons m ^-2^ s ^-1^, p< 0.05).

In summary, high maximum quantum yield of PSII, high thermal dissipation ability, low PSII quantum yield and low electron transport rate at light steps less than 147 μmol photons m ^-2^ s ^-1^ (p< 0.05) were observed for progenies #23 and #37. Progenies #18 and #19 revealed low photosynthetic efficiency and low electron transport rate at light steps less than 612 μmol photons m ^-2^ s ^-1^ (p< 0.05). Due to the low thermal dissipation ability at high levels of light steps (612–1077 μmol photons m ^-2^ s ^-1^, p< 0.05), progenies #18, # 19 and #20 showed stronger resistance to high PAR than the control group. The outcomes of chlorophyll fluorescence parameters indicate that the five progenies possess various characteristics of photosynthetic efficiency after carbon-ion exposure.

### Xanthophyll Cycle and Lutein Content of the Progenies after High Light Exposure

**Fig 5:**
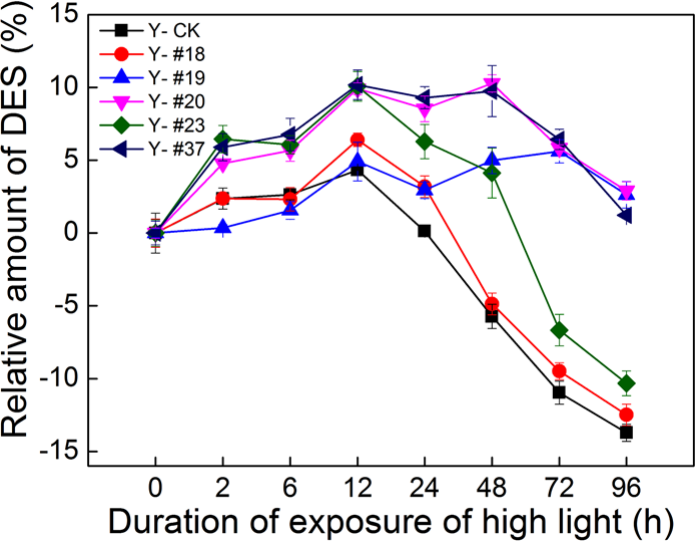
Relative amount of DES in the progenies and in the control group under HL exposure. The concentrations of Vx, Ax and Zx were measured by HPLC. *DES* = (*Zx*+0.5*Ax*)/(*Vx*+*Ax*+*Zx*). The five progenies and the control group were grown under HL exposure (300–400 μmol photons m^-2^ s^-1^) for 96 h. Each experimental HL exposure value was compared with the value found under normal conditions (0 h). n = 3+SD.

The de-epoxidation index in the xanthophyll cycle pool was calculated based on the equation of DES, which was measured by HPLC analysis [26] (Fig 5). During 12 h of high light (HL) treatment, the DES values of the five progenies and of the control group reached maximal values. DES values of progenies #19, #20 and #37 reached a plateau between 12 h and 48 h of HL exposure. The same trend in DES values found in the control group appeared in progenies #18 and #23. In contrast to the control group, progenies #19, #20, #23 and #37 showed a strong ability to dissipate excess energy as heat during 24–96 h HL exposure (p< 0.05).

**Fig 6:**
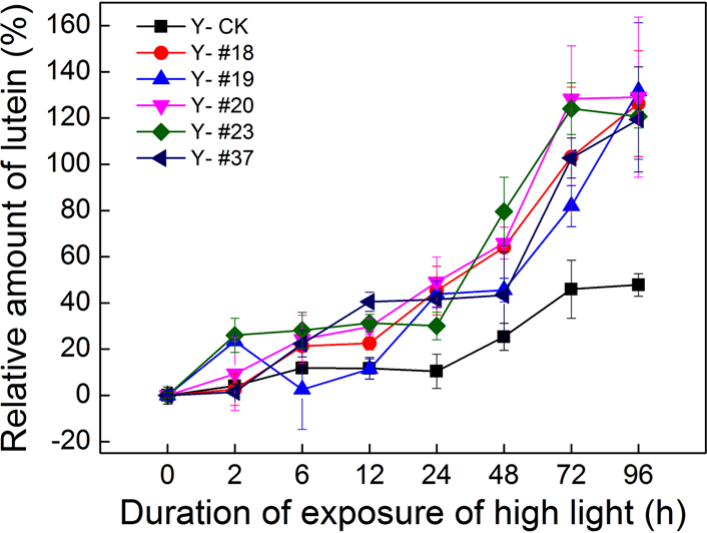
Relative amount of lutein in the progenies and in the control group during HL exposure. The concentration of lutein was measured by HPLC. The five progenies and the control group were grown under HL exposure (300–400 μmol photons m^-2^ s^-1^) for 96 h, and each value obtained during HL exposure was compared with that obtained under normal conditions (0 h). n = 3+SD.

Lutein is the most abundant carotenoid in the photosynthetic apparatus, and its occupancy is essential for protein folding and the quenching of ^3^Chl* [25]. The concentration of lutein in algal cells was measured by HPLC analysis (Fig 6). An increase in the lutein content of the five progenies and of the control group was found during 96 h of HL exposure. The lutein content of progenies #18, #20 and #23 increased markedly after 24 h of HL exposure (p< 0.05). This indicates that the three progenies possess a strong ability to quench ^3*^Chl under conditions of HL exposure (24 h-96 h). A striking augment of the lutein values in progenies #19 and #37 found at 96 h of HL treatment (p< 0.05). Thus, de-epoxidation of Zx and lutein content of the progenies #20 and #23 showed strong photoprotection ability after 24 h of HL exposure.

### Expression of Genes Existed in Light-Harvesting Complex II in the Progenies

**Fig 7:**
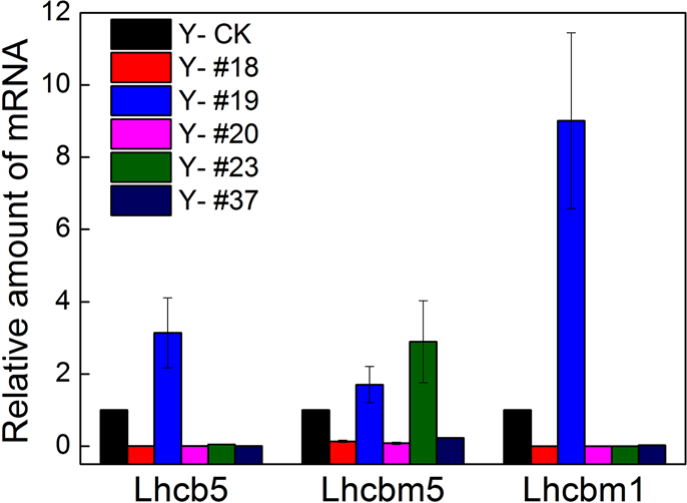
Expression of *Lhcb5*, *Lhcbm5 and Lhcbm1* genes in the five progenies. The levels of the indicated mRNAs in the five progenies were determined by qRT-PCR after 8 d of cultivation. The *18S* rRNA gene was used as a reference gene. n = 4+SD.

On exposure of plant cells to ionizing radiation, pigment-protein complexes including LHCII could be altered [8]. To test whether changes at transcriptional levels of LHCII occurred in the five progenies, the mRNA levels of three LHCII proteins (*Lhcb5*, *Lhcbm5* and *Lhcbm1*) were determined by qRT-PCR method (Fig 7). A markedly increased expression level in these three genes in progeny #19 compared with the expression of the control group (3.1-folds, 1.7-folds, and 9-folds) was observed. In progeny #23, the mRNA level of *Lhcbm5* was upregulated 2.8-folds, whereas the levels of *Lhcb5* and *Lhcbm1* mRNAs were downregulated to 0.05 and 0.001 compared with that of the control group (relative amount of transcript expression was 1), respectively. A large decreased expression level for the three genes in progeny #18 (downregulation to 0.007, 0.136, 0.001), #20 (0.004, 0.084, 0.006) and #37 (0.004, 0.23, 0.03) was observed.

## Discussion

### Carbon-Ion Irradiation and Pigment Progeny Screening

*Selenastrum capricornutum* showed higher sensitivity to carbon-ion irradiation compared with other microalgae. A 10% survival rate of *Selenastrum capricornutum* was observed after irradiation at 40 to 120 Gy; the five progenies studied in this work were all obtained from cultures irradiated above 90 Gy. These results indicate that a dose resulting in ~10% survival rate is suitable for mutation induction in microorganisms [27, 28, 29]. This viewpoint is also supported by the results of other studies. For instance, Hu, *et al*. isolated the high-lipid-productivity mutant D90G-19 from *Desmodesmus* sp. S1 after irradiation at 90 Gy with a 30% survival rate [13], and Ma, *et al*. obtained the high growth rate and high lipid productivity mutant HP-1 from *Nannochloropsis oceanica* IMET1 after irradiation at 160 Gy with a 10% survival rate [14]. Thus, mutants are frequently obtained from high lethal doses of radiation, and this could provide a reference irradiation dose for heavy-ion mutation induction and subsequent selection for breeding.

In photosynthesis, chlorophylls not only absorb light but also serve as electron donors and acceptors, mediating ultrafast energy transfer across the photosynthetic unit [30]. Chl *a*, which is more than other chlorophylls and is found in light-harvesting complexes antenna proteins as well as in the reaction centers of PSI and PSII [20]. Therefore, Chl *a* was chosen as our initial screening index. Our screening results showed that the Chl *a* content of all five progenies during the exponential growth phase was lower than that of the control group. This is in agreement with results reported by Angelini, *et al*., who stated that photosystems could be damaged by ionizing radiation [9]. Moreover, the observed dramatic reduction in chlorophyll content most likely reflected the altered expression of Chl-binding proteins [16]. Therefore, the amount of chlorophyll could be associated with the expression of related proteins and with the number of photosynthetic units. In addition, the predominant event of ionizing radiation is water radiolysis, which can induce oxidative damage by the generation of free radicals. Reactive oxygen species could result in over-reduction in the photosynthetic electron transport chain [8]. The resulting damage to energy transfer could lead to a reduction in the content of Chl *a*.

### Photosynthetic Efficiency of the Progenies under Normal Condition

Light energy is taken up by pigments, transformed into photosynthetic quanta, used in photosynthesis, and converted to chlorophyll fluorescence and thermal dissipation [23]. To further characterize the photosynthetic features of the five progenies obtained in this study, their chlorophyll fluorescence parameters were analyzed. According to the Fv/Fm results, progenies #23 and #37 revealed potentially high-efficiency utilization of a small amount of antenna pigment Chl *a* to improve the photosynthetic efficiency of PSII. The biomasses of progenies #23 and #37 have the same levels with that of the control group (shown in S1 Fig), which also verified the Fv/Fm results. Moreover, the two progenies showed weak adaptation in low PAR compared with the control group. This could be due to the low efficiency of electron transport rate, resulting in the conversion of less light energy during photochemistry reaction. At the same time, more thermal dissipation occurred in these progenies under conditions of excess excitation energy not used in photosynthesis. The different characteristics of the five progenies may be related to two important factors. One factor is that photosynthesis in the progenies is dynamically regulated by balancing energy distribution through regulating electron transport rate, thermal dissipation, etc. Another factor is that photosynthetic capacity could be improved through rearranging the size of the light-harvesting antenna, repairing mechanisms mediating PSII turnover, and altering the expression of genes for Chl-binding proteins and LHCs [31].

### Xanthophyll Cycle and Lutein after High Light Exposure and Expression of Genes in Light-Harvesting Complex II

De-epoxidation of Vx to Zx leads to enhanced dissipation of excess excitation energy in the PSII antenna system, thus preventing inactivation and damage to the photosynthetic apparatus [32]. The DES values of the five progenies and of the control group indicated that the five progenies adapted to the HL condition after 12 h of HL exposure. The enhancement of lutein content in the five progenies and in the control group indicated that additional ^3^*Chl was required for quenching, although all six strains adapted to long periods of HL exposure (> 12 h). The increase in Chl *a* content under HL exposure could provide an opportunity to generate more ^3^Chl^*^ (shown in S2 Fig). Thus, lutein appears to play a role in preventing excessive oxidation damage.

Progenies #20 and #23 showed stronger thermal dissipation ability and quenching of ^3^Chl^*^ than the control group after 24 h of HL exposure. This phenomenon was supported by the increased efficiency of a number of processes, including NPQ, ROS scavenging, ^3^Chl* yield and thylakoid membrane reorganization, all of which contributed to the increase resistance to excess light when they were upregulated by Zx accumulation. [26]. Baker, *et al*. reported that protonation of PsbS (LHCSR in algae) and binding of Zx to PSII, which produces conformational changes in the antennae, were the reason for the increased quantum yield of thermal dissipation of excitation processes [33]. Therefore, the strong thermal dissipation exhibited by the progenies could be caused by the expression of the Chl-binding proteins [34]. Murchie, *et al*. reported that excess light energy led to acidification of the thylakoid lumen due to accumulation of protons in the lumen through improvement in the xanthophyll cycle [16]. Thus, another possible reason for the increased thermal dissipation could be thylakoid membrane reorganization. According to the results of TEM micrographs (shown in S3 Fig), this possible reason for the photosynthetic characteristics of the progenies could be excluded. In addition, the expression of the genes encoding LHCII (*Lhcb5*, *Lhcbm5* and *Lhcbm1*) was analyzed. *Lhcbm1* encodes one of the most abundant LHCII proteins *Lhcb2*, and *Lhcbm5* encodes the major LHCII protein *Lhcb1*, which is present in the outer antenna layer [35–36]. *Lhcb5* encodes a minor LHCII protein that is present in the inner antenna layer [37]. The levels of mRNAs coding for these proteins in the five progenies verified that the markedly differences in the expression of LHCII proteins could be a major cause for the different photosynthetic features of the five progenies. For instance, upregulated mRNA levels of LHCII proteins in progeny #19 could result in low photosynthetic efficiency. Therefore, LHCII proteins could be a target for carbon-ion irradiation in photosynthetic system.

## Supporting Information

S1 Fig Biomass of the progenies and of the control group. (TIF)

S2 Fig Relative Chl *a* content of the progenies and of the control group after HL exposure. Each value obtained after HL exposure was compared with the value obtained under normal conditions (0 h). n = 3+SD.
(TIF)

S3 Fig TEM micrographs of the control group and of the progenies. Group A: control group; Group B: progeny #37; Group C: progeny #23; Group D: progeny #20. The micrographs show the cytoplasmic area with the chloroplast. A starch grain (①), plastoglobuli (②), and grana stacks (③) are indicated. Bars = 0.2 μm (column 2–3, column1 of A), 0.5 μm (column1of B-D).
(TIF)

S1 Table Screening of *Selenastrum capricornutum* progenies based on the content of Chl *a*. n = 3+SD. ^a^ decreased relative to the control group (p < 0.05).
(PDF)
