# Supplementary figures and images for: Photosynthetic Effect in Selenastrum capricornutum Progeny after Carbon-Ion Irradiation
Source: PLoS One. 2016 Feb 26;11(2):e0149381. doi: 10.1371/journal.pone.0149381 (PMC4769097; doi:10.1371/journal.pone.0149381)

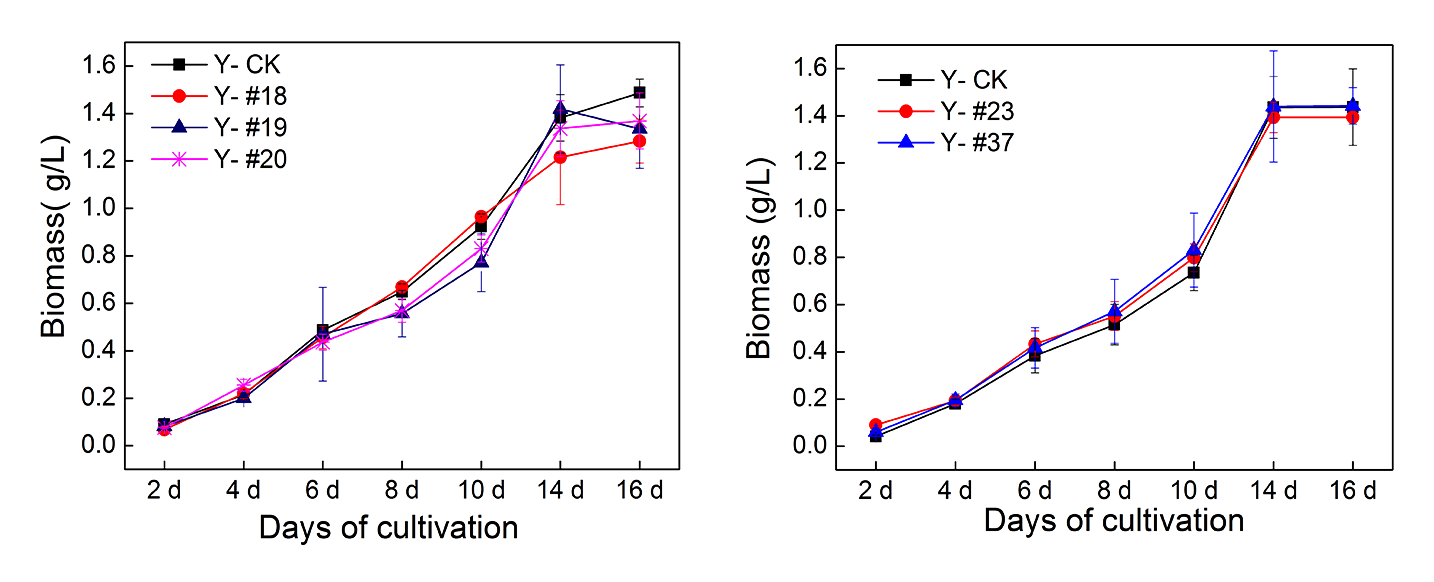

Supplement: S1 Fig — (TIF) [file pone.0149381.s001.tif]

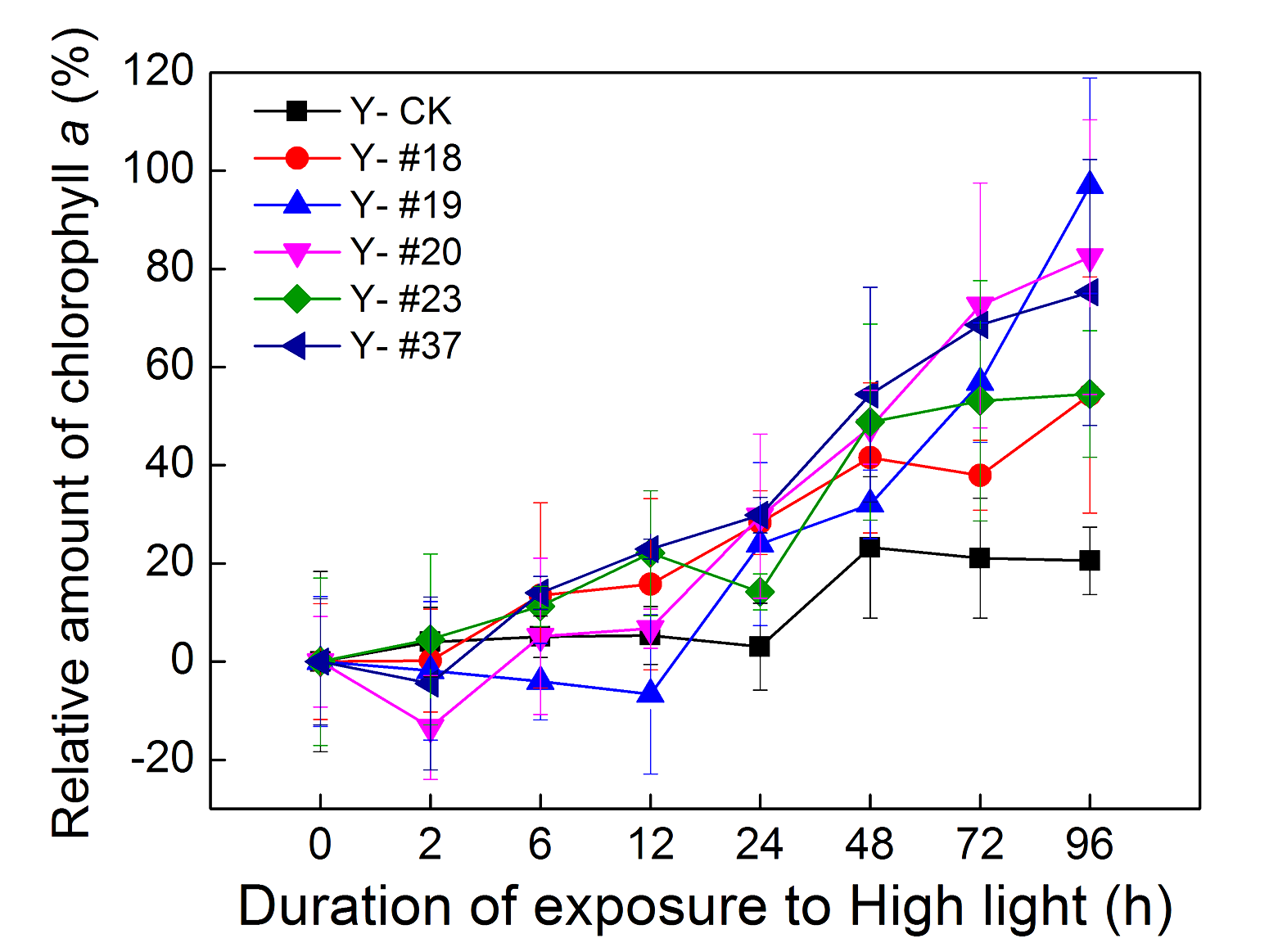

Supplement: S2 Fig — Each value obtained after HL exposure was compared with the value obtained under normal conditions (0 h). n = 3+SD. (TIF) [file pone.0149381.s002.tif]

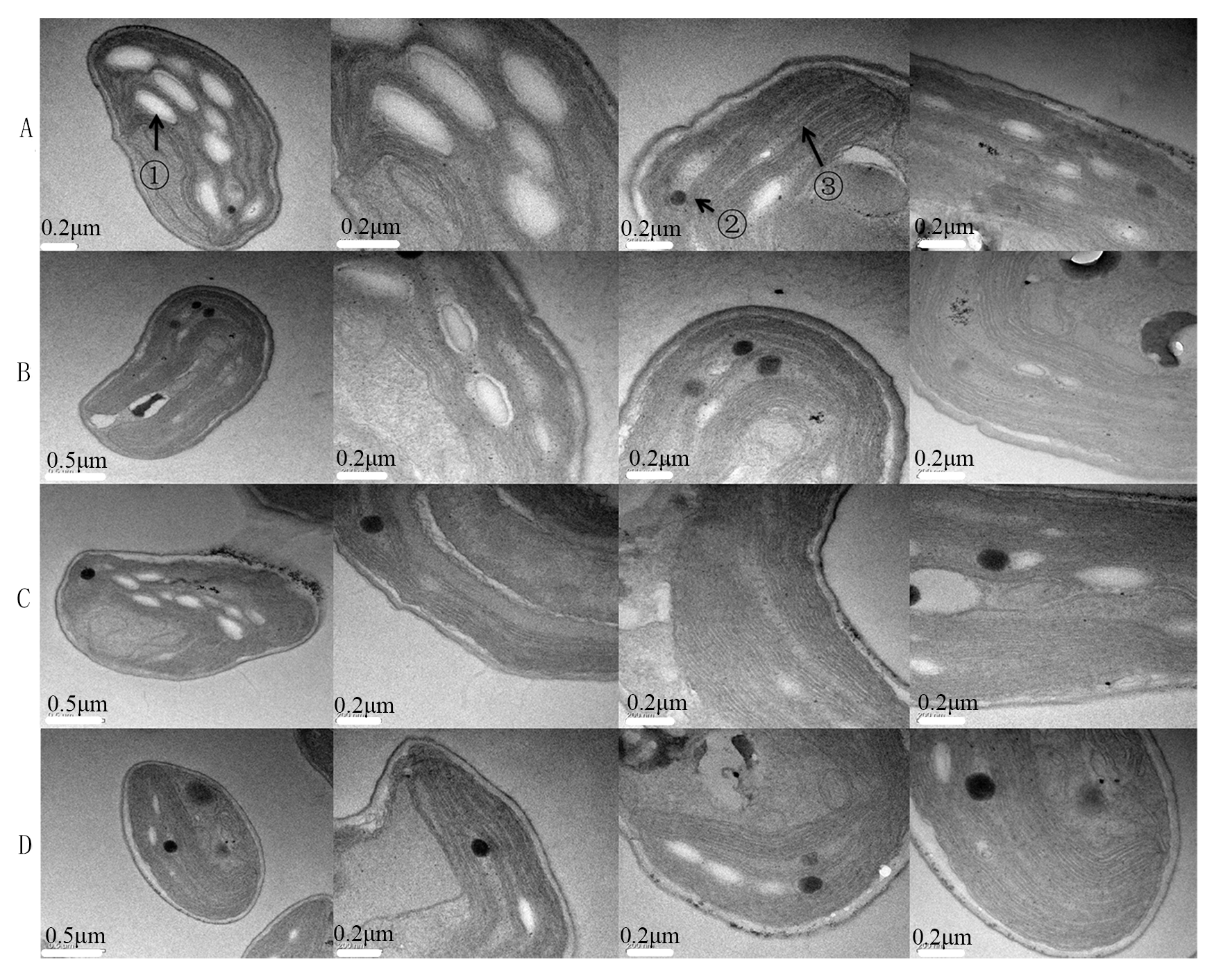

Supplement: S3 Fig — Group A: control group; Group B: progeny #37; Group C: progeny #23; Group D: progeny #20. The micrographs show the cytoplasmic area with the chloroplast. A starch grain (①), plastoglobuli (②), and grana stacks (③) are indicated. Bars = 0.2 μm (column 2–3, column1 of A), 0.5 μm (column1of B-D). (TIF) [file pone.0149381.s003.tif]
