# Supplementary material for: Photosynthetic Effect in Selenastrum capricornutum Progeny after Carbon-Ion Irradiation
Source: PLoS One. 2016 Feb 26;11(2):e0149381. doi: 10.1371/journal.pone.0149381 (PMC4769097; doi:10.1371/journal.pone.0149381)
Supplement: S1 Table — a decreased relative to the control group (p < 0.05). (PDF) [file pone.0149381.s004.pdf]

Chl a (μ g/L)	Y- CK	Y- #18	Y- #19	Y- #20	Y- #23	Y- #37
**2 d**	63.58 ±0.98	29.92 ±1.89[a]	41.29 ±10.9013[a]	30.93 ±3.02[a]	34.82 ±4.81[a]	52.85 ±6.17[a]
**4 d**	86.86 ±0.13	55.23 ±4.11[a]	50. 90 ±13.29[a]	50.40 ±10.72[a]	59.21 ±6.46[a]	72.53 ±6.13
**6 d**	99.59 ±6.74	64.45 ±8.50[a]	58.97 ±12.04[a]	71.38 ±7.01[a]	73.85 ±0.80[a]	79.78 ±2.62[a]
**8 d**	123.47 ±9.95	82.89 ±15.36[a]	69.15 ±4.40[a]	92.74 ±13.39[a]	89.38 ±6.41[a]	117.70 ±4.10
**10 d**	153.19 ±9.89	115.95 ±11.04[a]	116.92 ±6.31[a]	115.71 ±2.41[a]	118.49 ±6.46[a]	120.64 ±4.26[a]
